# Supplementary figures and images for: Spatiotemporal Coding in the Macaque Supplementary Eye Fields: Landmark Influence in the Target-to-Gaze Transformation
Source: eNeuro. 2021 Jan 21;8(1):ENEURO.0446-20.2020. doi: 10.1523/ENEURO.0446-20.2020 (PMC7877461; doi:10.1523/ENEURO.0446-20.2020)

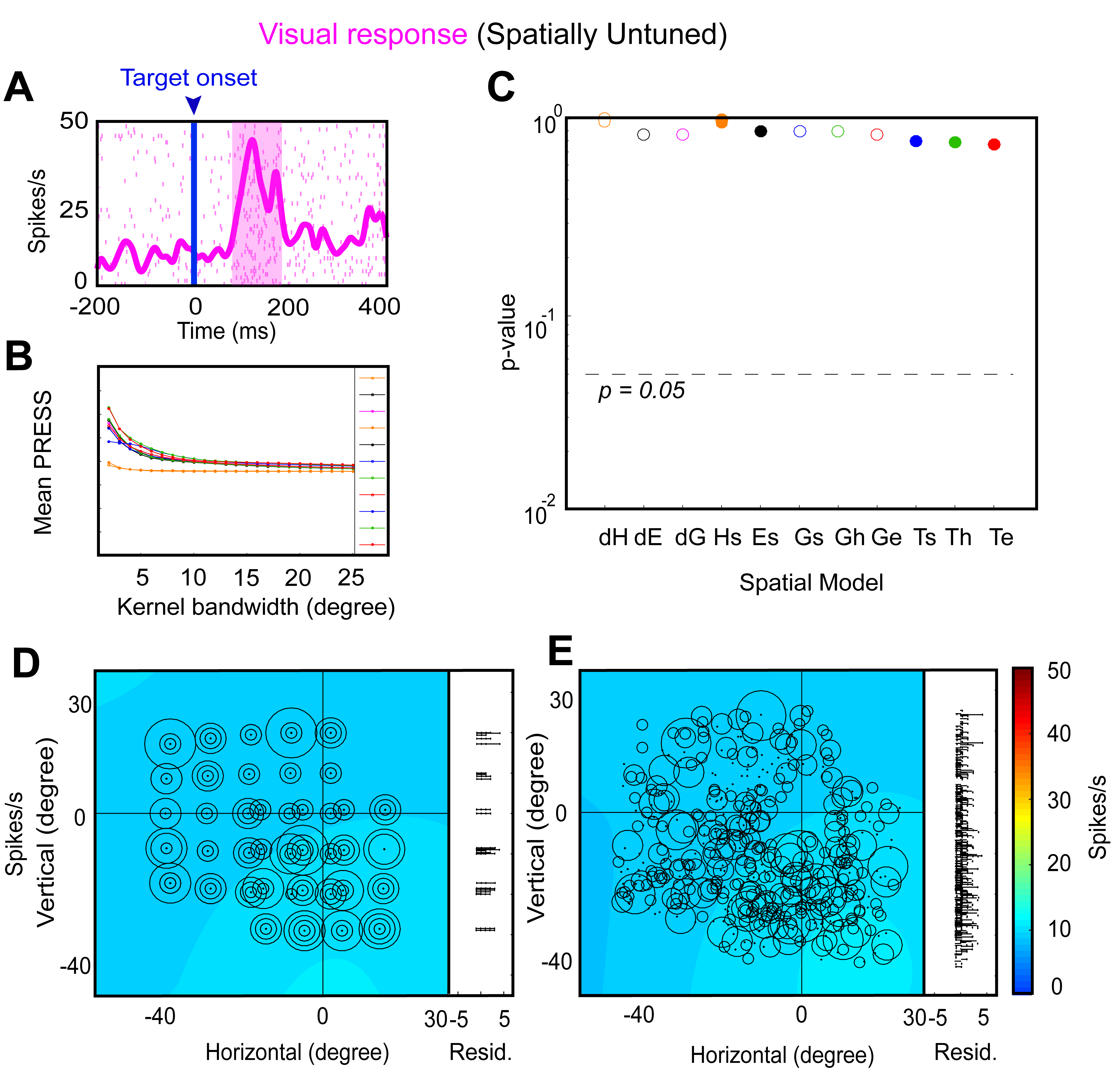

Supplement: Extended Data Figure 5-1 — An example of a spatially untuned visual neuron. A, Raster/spike density plot (with top 10% responses) of the visual neuron aligned to the onset of target (blue arrow); the shaded pink region corresponds to the sampling window (80–180 ms) for response field analysis. B, Mean residuals from the PRESS-statistics for all spatial models at different kernel bandwidths (2–25°). C, The p values statistics and comparison between different models. No model was significantly eliminated. D, Representation of neural activity for Ts: target in space (screen). The circle corresponds to the magnitude of the response, and heat map represents the non-parametric fit to these data. The corresponding residuals are displayed to the right. E, Representation of the neural activity in Te (target in eye). Download Figure 5-1, TIF file. [file enu-eN-NWR-0446-20-s01.tif]

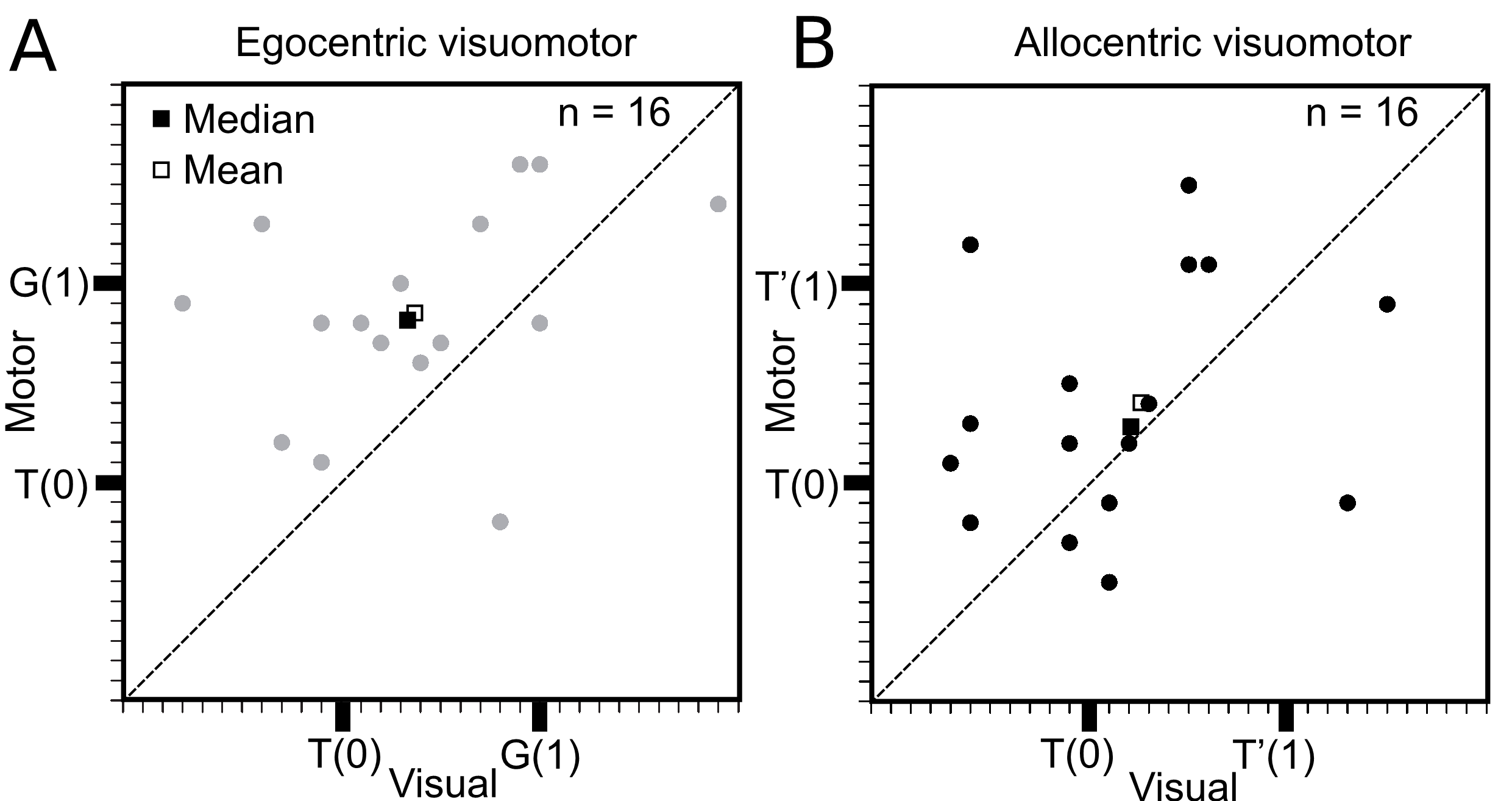

Supplement: Extended Data Figure 10-1 — VM transformation at the single cell level. A, Significant visual to motor transformation within the VM neurons along the T-G continuum. B, No significant visual to motor transformation along the TT’ continuum. Download Figure 10-1, TIF file. [file enu-eN-NWR-0446-20-s02.tif]

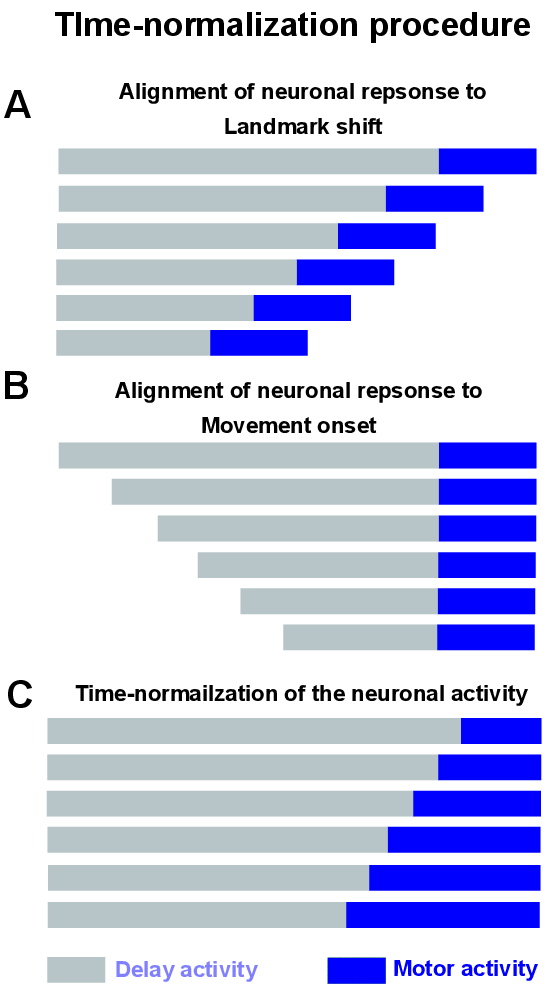

Supplement: Extended Data Figure 11-1 — Time normalization procedure. A, Alignment of responses to the landmark shift. B, Alignment of responses to the saccade onset. Note: the alignment of responses in the standard was as in A, B leads to loss and/or mixing of responses thus not allowing us to track spatial codes through the entire trial across all trials. C, Time normalization from landmark shift until saccade onset. This procedure where we divide the activity into equal half-overlapping bins across all trials allows us to treat all the trials equally, thus as a continuum. Download Figure 11-1, TIF file. [file enu-eN-NWR-0446-20-s03.tif]
